# Spatial Olfactory Memory and Spatial Olfactory Navigation, Assessed with a Variant of Corsi Test, Is Modulated by Gender and Sporty Activity

**DOI:** 10.3390/brainsci12081108

**Published:** 2022-08-19

**Authors:** Sara Invitto, Giuseppe Accogli, Mariangela Leucci, Marika Salonna, Tonia Serio, Francesca Fancello, Vincenzo Ciccarese, Dion Lankford

**Affiliations:** 1 Inspire Lab, Laboratory of Cognitive and Psychophysiological Olfactory Processes, Department of Biological and Environmental Sciences and Technologies, University of Salento, 73100 Lecce, Italy; 2 Istituto Santa Chiara, 73100 Lecce, Italy

**Keywords:** spatial olfactory navigation, spatial representation, spatial memory, olfaction, physical activity, sport, Corsi Block Tapping Test

## Abstract

Many studies have focused on navigation, spatial skills, and the olfactory system in comparative models, including those concerning the relationship between them and physical activity. Although the results are often in contrast with each other, it is assumed that physical activity can affect cognition in different ways—both indirectly and through a certain influence on some brain structures. In contrast, there is little research that focuses on the relationship between spatial abilities and olfactory abilities in humans. This research aimed to evaluate and compare the performance in working memory tasks of athletes and non-athletes who require good visual–spatial navigation, olfactory–spatial navigation, and olfactory–semantic skills. The study involved 236 participants (83 athletes) between the ages of 18 and 40. All subjects were matched by age or sex. The standard Corsi Block Tapping Test (CBTT) was administrated to investigate the visual-spatial memory. Olfactory–spatial navigation and olfactory–semantic skills were assessed with two modified versions of CBTT: Olfactory CBTT (OCBTT) and Semantic–Olfactory CBTT (SOCBTT) respectively. The results show differences between the CORSI conditions in direction of a poor performance for athletes. A gender effect in favor of men was also found, particularly in the classic version of the CBTT. Both groups performed better in the classic version of the CBTT than OCBTT and SOCBTT. The mean of SOCBTT results is markedly lower, perhaps due to the different information processing systems needed to perform this kind of task. It is possible to explain how sports practice can affect tasks that require spatial skills and olfactory perception differently, thus supporting new hypotheses and opening new scientific horizons.

## 1. Introduction

### 1.1. Human Spatial Representation and Navigation

In humans, spatial navigation is generally regulated by three types of spatial knowledge. It is possible to distinguish between: landmarks, i.e., fixed salient features or points of reference in the environment; route knowledge, i.e., sequences of locations as experienced by the navigator, and thus associated with an egocentric reference system; survey knowledge that includes information about the general structures of routes and the spatial relationships between different sites and associated with an allocentric reference system; graph knowledge, a network of maps and places that consists of a topological connection between various locations [1,2,3,4]. Moreover, there are three strategies used in space navigation: egocentric, allocentric, and beacon. The allocentric representation (world-focused) refers to landmarks external to the navigator, while the egocentric type of representation (body-focused) implies a reference to the current body position of the individual. Beacon concerns navigation to one or more objects and requires the memory of the object itself and the ability to distinguish it from other objects and characteristics; therefore, it could be considered as an object that indicates a nearby target location or the target itself [2,5].

A neural network underlying space navigation in humans is based both on specific cells activated in response to specific spatial positions—mainly present in the hippocampal cells that respond to the sight of landmarks. The hippocampus blends spatial and visual characteristics with the context to calculate flexible representations that are similar to maps of space [6]. The hippocampus, in addition to being involved in the organization and expression of memories, is essential for space navigation [7].

In particular, the hippocampus and entorhinal cortex could contain map-like spatial codes, while the parahippocampal and retrosplenial cortex could provide the inputs that allow for cognitive maps to connect to fixed environmental reference points [8,9].

Jacobs et al. [10] have identified cells in humans with a similar activity to grid cells, which are believed to be responsible, in animals, for numerous spatial behaviors. These are mainly distributed in the entorhinal and cingulate cortices and the hippocampus.

In addition, right-lateralized brain activities have been identified in spatial navigation tasks. This allowed for us to hypothesize that gamma oscillations in cerebral electrical activity in the right neocortex (in particular, in the temporal, parietal, and occipital cortices) play an important role in human space navigation [11].

Furthermore, human space navigation would also seem to be influenced by age and sex [12,13,14].

### 1.2. Spatial Navigation and Olfaction in Humans

Jacobs [15] hypothesizes that the primary function of olfaction was navigation, thanks to its ability to map odorants in space, as well as to discriminate them.

In the animal kingdom, the sense of smell still plays a vital role in navigation and spatial orientation. A hippocampal region associated with spatial orientation and an olfactory–hippocampal projection is preserved in some groups of animals, such as rodents, in which the olfactory system is connected to the hippocampus through the entorhinal cortex [16]. With regard to the spatial organization, young rats are better at using the sense of smell but are less efficient at using visual information as opposed to older rats, although there is still an interaction between different sensory modalities [17]. Seabirds are able to use olfactory cues to navigate even in very large spaces, as well as to search for food [18].

However, few studies focus on the role of smell in human spatial navigation. For example, Bao et al. [19] explain how odor information can be assembled into spatially navigable cognitive maps, optimizing orientation, and pathfinding to an odor source. Dahmani et al. [20] show that particular structures of the human hippocampus (specifically the fimbria–fornix volume) are connected to both navigational learning and olfactory identification. Dahmani et al. [21] focus on how olfactory identification covaries with spatial memory and how the thickness of the left medial orbitofrontal cortex and the volume of the right hippocampus predict both olfactory identification and spatial memory. Hamburger and Knauff [22] show how humans are able to expand their cognitive map of the environment with olfactory landmarks used in spatial orientation.

Jacobs et al. [23] show that humans can use the olfactory modality to map and reorient themselves in a previously learned place. Goodrich-Hunsaker et al. [24] found that the hippocampus is essential for associative odor-place memory and spatial recognition memory, supporting the hypothesis that associative odor–place memory is mediated by the hippocampus in both rodents and humans.

This highlights that there is a lack of information regarding the connection between the olfactory modality, orientation, and spatial navigation in humans.

### 1.3. Spatial Navigation in Athletes and Non-Athletes

Spatial representation and navigation represent one of the many cognitive abilities available, and therefore they can influence human life in many contexts. In the recent literature, many studies focus on the relationship between physical activity and its impact on human cognitive abilities—including representation and space navigation—even if there is no unanimous agreement regarding the benefits of physical activity on the cognitive system [25,26].

Although a specific and clear link has not yet been identified, it is assumed that physical exercise may increase cognition indirectly, for example, by improving health and reducing chronic diseases that have a certain impact on neurocognitive functions [27,28]. Furthermore, the benefits associated with physical activity may vary based on genetic or diet-related factors [29].

Some studies report a strong influence of physical activity on brain functions—such as learning, memory, executive functions— and cognitive decline [30,31], as well as an increased volume of gray and white matter regions [32] in the hippocampus. High levels of aerobic exercise appear to be associated with a more extensive right and left hippocampus, and larger hippocampus and higher fitness levels seem to be correlated with improved spatial memory performance [33,34].

Moreover, increased physical activity appears to be linked to greater hippocampal and basal ganglia volume, greater white matter integrity in preadolescents, and greater volumes of the prefrontal cortex and basal ganglia, as well as the hippocampus, in older adults [29].

In addition, physical activity has a more significant effect on cognitive functioning and the future incidence of cognitive decline among subjects carrying at least one copy of the APOE ε4 allele [35]. It could represent an advantage in reducing the risk of Alzheimer’s disease, other forms of dementia (excluding vascular dementia), and cognitive decline [36].

There are not many studies that specifically focus on the relationship between spatial ability and athletes, I and results are often in contrast with what has been previously reported. For example, Cynthia et al. [37] reported that the spatial ability of athletes and non-athletes is not significantly different from each other, concluding that exercise may or may not increase the spatial capacity of both groups. However, specific sport skills did not further affect the spatial abilities of the athletes. Jansen and Lehmann [38] examined visual–spatial cognition in athletes and non-athletes through an object-based mental rotation task, showing that all participants had greater accuracy for the rotation of human figures than objects and only one class of the athletes considered demonstrated a better mental rotation performance than non-athletes.

### 1.4. Visual–Spatial Memory and Olfaction

The literature shows that olfaction may be related to tasks that require the use of visual–spatial memory [39,40,41].

Furthermore, some researchers hypothesized that olfactory identification and visuospatial memory would be linked by overlapping brain areas, which include the left orbitofrontal cortex and the right hippocampus [21]. Moreover, the visual–spatial component is also extremely present in the olfactory perception, where, from an evolutionary point of view, the further development of ancestral olfactory abilities is linked to the location of the olfactory marker. Evolution has favored the development of vision, leading to a decline in the olfactory sense in the human being [42].

Recently, research has focused on the link between olfactory stimuli and cognitive and physical performance in athletes [23,43,44].

For this reason, this study aimed to examine and compare the performance of athletic subjects with non-athletic subjects in working memory tasks that require good visual–spatial navigation skills, olfactory–spatial navigation skills, and olfactory–semantics skills.

## 2. Materials and Methods

The Corsi Block Tapping Test (CBTT) associated with olfactory components was used to investigate how sense of smell can be related to tasks requiring the use of visuo-spatial memory, and whether this component could be gender-dependent. For this reason, the possibility of a gender difference was also considered.

The study took place at the Laboratory of Cognitive and Psychophysiological Olfactory Processes (INSPIRE) at the University of Salento, Lecce. The experimental procedure of the study was approved by the Ethical Committee (IRB) of the DiSTeBA, University of Salento. All participants signed a written informed consent form before inclusion in the study.

### 2.1. Participants

The research involved 236 volunteer subjects (aged between 18 and 40 years), who were recruited, assessed, and selected without allergies or evident respiratory/olfactory alterations. They were divided as follows: 83 athletic subjects (group 1), enrolled in the department of Motor Science (54 men; mean age 22.4 ± 5.5) and 153 non-athletic subjects (group 2), enrolled in the department of psychology (61 men; mean age 22.6 ± 4.3). The athletes were recruited from students recognized as student-athletes, belonging to different competitive sports categories (including football, judo, martial arts, horse riding, swimming, basketball, and boxing). A specific sport was not chosen for this study because, as already highlighted in the introduction, specific sport skills did not further affect the spatial abilities of the athletes [37,38]. The control group included non-athlete subjects, who did not follow competitive activities or participate in sports activities for more than two hours a week.

### 2.2. Olfactory Stimuli

Olfactory Stimuli were purchased as pure odorants by Sigma-Aldrich Products.

Table 1 shows the chemicals used and the common names associated with the odorants.

The odorants were taken pure with a 5 mL syringe, and, for each square of tissue paper, 3 mL of odorant was instilled. Each set of odorants was placed in a transparent envelope numbered with the number associated with the Corsi block tapping test.

### 2.3. Assessment

The CBTT [45,46] was used to examine visual–spatial memory. It consisted of nine blocks arranged irregularly on a 23 × 28 cm board [47]. Each cube was numbered on the side facing the examiner to simplify the execution.

The CBTT was administered according to three different sessions and modalities: (1) classical version of the CBTT; (2) Olfactory CBTT (OCBTT); and (3) Semantic-Olfactory CBTT (SOCBTT).

In the classical version (CBTT) [48], the examiner (E) showed the subject (S) a spatial sequence by touching the numbered cubes at a regular interval of about 2 s.

The S was seated in front of the E. The E told the S to “touch the cubes that I touch, immediately after me” and touched cube number 1 with their index finger, before returning it to the table placed between himself/herself and the S each time. The S’s task was to touch the same cube with the dominant hand. This continued, in order, for all cubes up to number 9. If the subject did not make any mistakes, the test is carried out, otherwise the preliminary test was repeated up to a maximum of 3 times.

The E touched the index sequences of cubes and progressively increased the length (from 2 to 10 cubes) at the rate of one cube every 2 s, returning the index finger to the table after of each touch. As soon as the demonstration of the sequence finished, the E asked the S to reproduce it by noting the cubes in the same order. Three sequences were presented for each series. If the S correctly reproduced at least 2 out of 3 sequences, the next series was examined [45] (see Video S1 CBTT).

**Figure 1:**
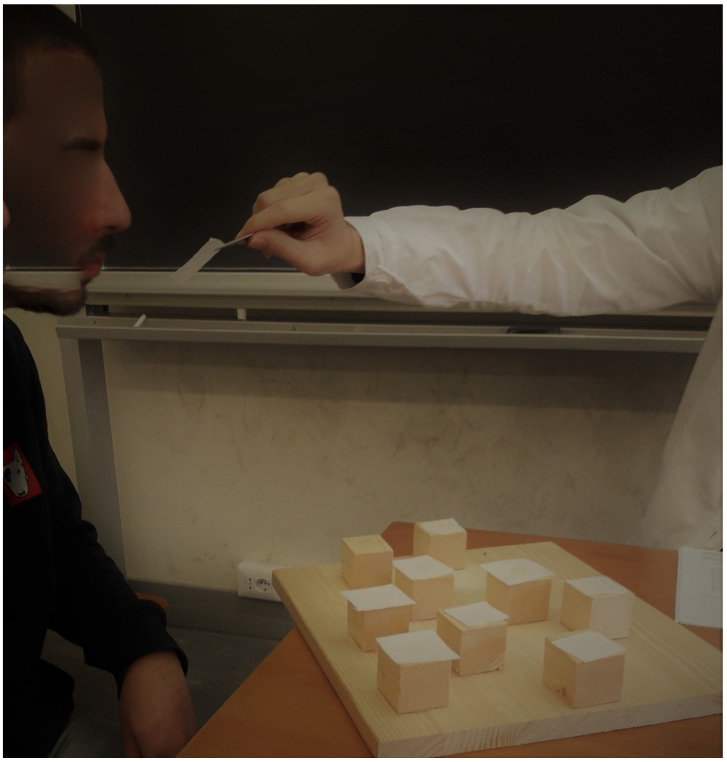
Example of the Olfactory Corsi Block Tapping Test (OCBTT).

The OCBTT is an experimental and innovative version of the CBTT. In OCBTT, squares of paper wet with specific odorants were placed on the cubes. These squared papers were smelled according to sequences and methods described for the standard CBTT (see Figure 1 and Video S2  OCBTT).

The position of the blocks, the same for all subjects, was described in the Table 1.

The individual blocks of tissue paper with odorants were placed on the individual blocks and were subsequently picked up by the experimenter with long tweezers. At the end of each sequence, the odorant paper square, pseudo-randomly chosen, was extracted from a plastic pocket, and administered to the subjects. The subjects had to link the cube to the odorant they had just smelled during the sequence’s administration. In this version, the test was considered complete after three consecutive errors (see Videos S1 and S2).

In the SOCBTT, the examiner read the complete list of odorants used only once. Then the subjects performed the same task as the OCBTT, but, at the end of each sequence, the subject had to name the recognized odorants during the administration of the sequence and identify their relative position on the cubes (see Video S3 SOCBTT).

The tests’ sequence was alternated for each subject to avoid a primacy and habituation effect linked to olfactory stimulation.

### 2.4. Statistical Analysis

SPSS software was used for statistical data analysis. A GLM repeated measures was performed, considering the three variants of the Corsi Test as within condition (3 levels: CBTT; OCBTT; SOCBTT) and the SPORT as between condition (athletes, non-athletes), also considering sex and smoking as covariate.

## 3. Results

The descriptive analysis for each version of the test in each group showed the following results:

CBTT (group 1: mean 5.30; SD 0.852; group 2: mean 5.574; SD 1.061), OCBTT (group 1: mean 3.37; SD 2.105/group 2: mean 4.61; SD 2.191), SOCBTT (group 1: mean 0.51; SD 1.713/group 2: mean 3.22; SD 3.056).

**Figure 2:**
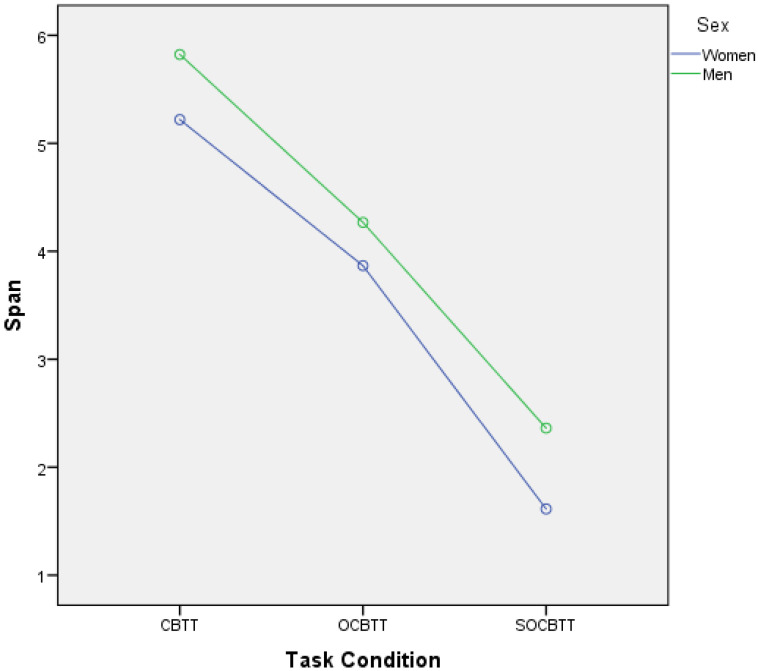
Gender difference and span expressed in the different task conditions (i.e., CBTT; OCBTT and SOCBTT).

**Figure 3:**
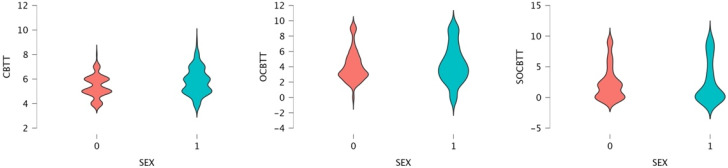
Violin Plot for gender difference and Span expressed in CBTT, OCBTT and SOCBTT.

**Figure 4:**
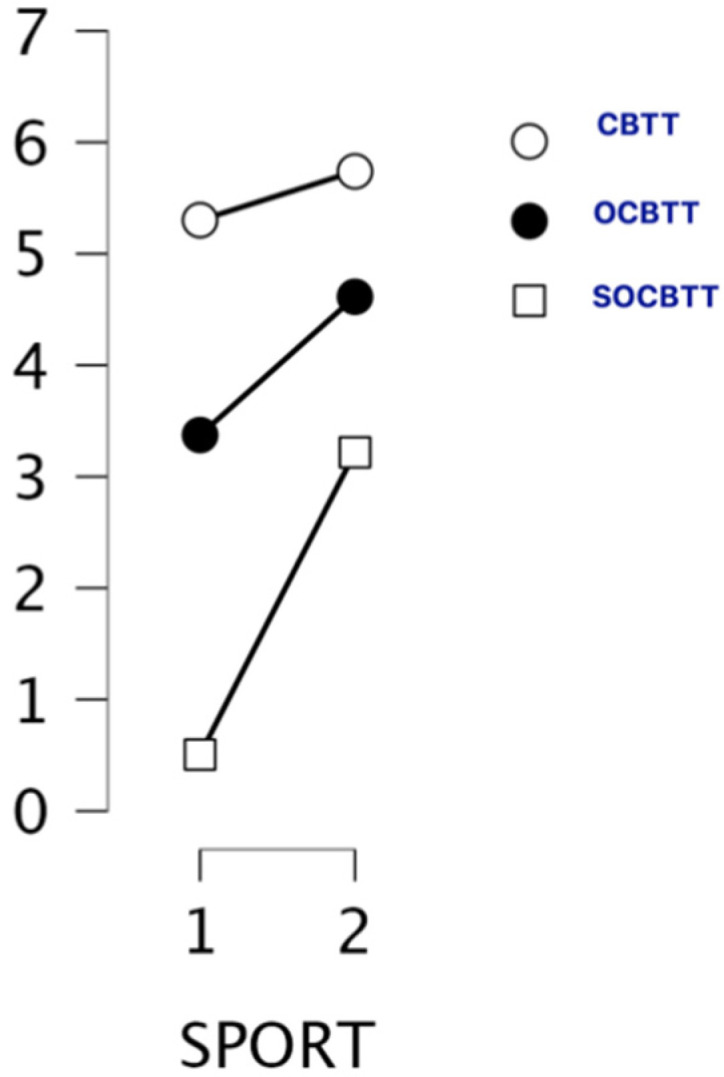
Span and sport conditions expressed through the different task conditions (i.e., CBTT; OCBTT and SOCBTT).

**Figure 5:**
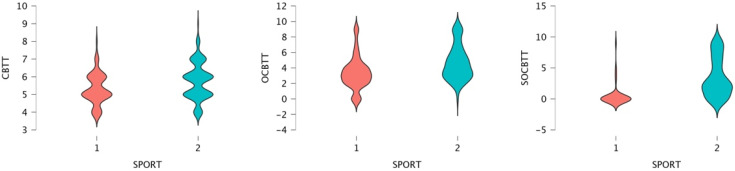
Violin plot for the span and sport conditions (1 = athletes; 2 = non-athletes) through the CBTT, OCBTT and SOCBTT.

A GLM repeated-measures showed, for the variable SPORT (athletes/non athletes) on TEST (3 Levels), a significant value on CBTT (*p* = 0.000; F = 16.055 part η^2^ = 0.075), on OBTT (*p* = 0.000; F = 16.331; part η^2^ = 0.076) and on SOBTT (*p* = 0.000; F = 52.204; part η^2^ = 0.209). Moreover, the results highlighted a significant effect for smoke in SOBCTT (*p* = 0.002; F = 10.252; part η^2^ = 0.049) and for sex in CBTT (*p* = 0.000; F = 18.154; part η^2^ = 0.084) and in SOBCTT (*p* = 0.006; F = 7.87; part η^2^ = 0.038) (Figure 2 and Figure 3). Multivariate test highlighted significant value for Factor 1 (*p* = 0.000; F = 113.92; part η^2^ = 0.586); significant interaction between Factor 1 and smoke (*p* = 0.003; F = 6.144; part η^2^ = 0.141) and significant interaction between Factor1 and SPORT (*p* = 0.000; F= 16.396; part η^2^ = 0.141) (Figure 4 and Figure 5).

Both groups (athletes and non-athletes) performed better on the CBTT (mean scores for athletes 5.30; mean scores for non-athletes 5.74). Both groups showed lower performance on the SOCBTT (mean scores for athletes 0.51; mean scores for non-athletes 3.22).

In general, male and female non-athletes showed a better performance on the classic version of the Corsi Test than athletes (CBTT athletes 5.30; CBTT non-athletes 5.74) (see Figure 2 and Figure 3).

## 4. Discussion and Conclusions

The olfactory component can be fundamental if we consider spatial navigation and spatial memory. The subcortical pathways that represent these aspects are connected to hippocampal and entorhinal activation, where, the chemoceptive component is relevant [49]. The recent literature has focused on the link between olfactory stimuli and cognitive and physical performance in athletes [23,43,44], highlighting no specific differences between the types of sport, but intensive or competitive athletic activity can be considered as a non-specific sport clustering factor.

For this reason, this study aimed to examine and compare the performance of athletic subjects with non-athletic subjects in working memory tasks that require good visual–spatial navigation skills, olfactory–spatial navigation skills, and olfactory–semantics skills, using a modified paradigm linked to the classical CBTT. This aspect can be a limit, but also a strength of the work, as it proposes a new task, connected to a classic one, and integrates an olfactory sensescape that could be strongly suitable for (but currently missing from) neuropsychological testing [50].

The main results of this study highlighted significant differences between various levels of the task (CBTT, OCBTT, SOCBTT) for the SPORT variable, and for this reason, lower scores are noted for athletes, which indicates a better performance on tasks for non-athletes. Furthermore, it is possible to observe a positive correlation between the variants of the tasks and the sports practice.

Both groups (athletes and non-athletes) had a better performance in the classic version of the CBTT than the other two variants (i.e., OCBTT and SOCBTT). The mean of the SOCBTT results is markedly lower than the classic variant of the CBTT and the OCBTT, possibly because it may be more difficult to perform the task due to the different information processing systems required.

As reported in the literature [13,14], in this study the gender effect was also confirmed. Men in both groups performed better than women in the classic variant of the CBTT and in the SOCBTT, while no significant difference was found in the OCBTT. More specifically, it is possible to observe that non-athlete men and women perform better in carrying out the task than men and women athletes. The lower performance of athletes was a result, although not hoped for, that was partly present in the literature [51]. The question of whether athletes have greater cognitive performance than controls has been much debated [52].

A limitation of the study was not considering the frequency of use of words connected to odorants, their familiarity, or their pleasantness. These variables may affect memory and recall during the task [50].

It is possible to explain how sports practice can differently effect tasks that require the use of spatial skills and olfactory perception, thus supporting new hypotheses.

This research reveals some prospects. Indeed, it may be appropriate to analyze whether and how sports activity frequency can influence olfactory perception and spatial memory tasks. In future, we will evaluate these variants of the test with a behavioral and psychophysiological analysis in clinical aging (e.g., MCI), where spatial and olfactory abilities are impaired [53], during rehabilitative motor activity, and through the use of an olfactometer also interfaced in EEG.

## Figures and Tables

**Table 1:** Name of the chemical/odorant used and common parfum name associated to the odorant.

Odorant	Common Parfum Name	Corsi Block Number
Acetophenone	Solvent	8
Carvone	Mint	2
Cinnamaldehyde	Cinnamon	9
Eucalyptol	Eucalyptus	1
Eugenol	Cloves	3
Geraniol	Geranium	5
Hexanal	Grass	7
Isoamyl Acetate	Banana	4
Phenethyl Alcohol	Rose	6

## Data Availability

The data presented in this study are available on request from the corresponding author sara.invitto@unisalento.it.

## Supplementary Materials

The following supporting information can be downloaded at: https://www.mdpi.com/article/10.3390/brainsci12081108/s1, Video S1: CBTT; Video S2: OCBTT; Video S3: SOCBTT.
